# *Helicobacter pylori* Infection Is Associated With Thyroid Dysfunction in Children With Congenital Hypothyroidism

**DOI:** 10.3389/fped.2022.875232

**Published:** 2022-06-21

**Authors:** Ivani Novato Silva, Lara Vieira Marçal, Dulciene Maria Magalhães Queiroz

**Affiliations:** ^1^Pediatrics Department, Faculdade de Medicina, Universidade Federal de Minas Gerais, Belo Horizonte, Brazil; ^2^Laboratory of Research in Bacteriology, Faculdade de Medicina, Universidade Federal de Minas Gerais, Belo Horizonte, Brazil

**Keywords:** *Helicobacter pylori*, congenital hypothyroidism, thyroiditis autoimmune, primary hypothyroidism, childhood, triiodothyronine

## Abstract

*Helicobacter pylori* (*H. pylori*) infection leads to a systemic low-grade inflammatory state and has been associated causally with a diverse spectrum of extra-gastric disorders. Among them, the infection has been involved in the pathogenesis of autoimmune thyroid disease (ATD), but only one study had evaluated children. Therefore, a cross-sectional study was conducted in a cohort of 142 children and adolescents, randomly assessed among those followed up for thyroid diseases in a university pediatric endocrinology service: 106 with congenital hypothyroidism (CH) and 36 with ATD. All children were asymptomatic, under strict control on levothyroxine replacement, and reported no other diseases or use of drugs. *Helicobacter pylori* status was evaluated by the ^13^C-Urea Breath Test (^13^C-UBT). Antithyroid antibodies (ATPO, antiTg, and TRAb) and serum thyroid hormones (TSH, free T4, and T3) were assessed by standard assays. Data were analyzed in logistic models by the SPSS statistical software package, and a *p*-value ≤ 0.05 was considered statistically significant. The prevalence of *H. pylori* infection was 19.44% in children with ATD. Neither the gender nor the serum levels of thyroid hormones and antithyroid antibodies were associated with the *H. pylori*-positive status. Thirty-seven (34.90%) children with CH were infected with *H. pylori*. The mean T3 serum level (3.59 ± 0.84) was significantly lower (*p* = 0.001) in the infected children than in those free from the infection (3.95 ± 0.89), association that remained after adjustment for the other variables in the multivariate analysis. Because no difference was observed in the levels of TSH and T4, the results indicate that the infection may lead to impairment in the thyroid hormonal balance, but not in the hypothalamic-pituitary-thyroid axis function. In as much as *H. pylori* infection is highly widespread and the prevalence of CH is also not negligible, additional studies are required to confirm our results and to identify the involved mechanisms.

## Introduction

*Helicobacter pylori* (*H. pylori*) is recognized as the main etiological agent of gastritis in human beings and as an essential factor in the pathogenesis of peptic ulcer, gastric carcinoma, and mucosa-associated lymphoid tissue (MALT) type gastric lymphoma (1).

Furthermore, *H. pylori* infection has been associated causally with a diverse spectrum of extra-gastric disorders including iron deficiency anemia, chronic immune thrombocytopenic purpura, growth retardation, and diabetes mellitus (2).

The infection may also be involved in the pathogenesis of autoimmune thyroid disease (ATD), including Hashimoto thyroiditis (HT), and Graves' disease (GD), the major causes of hypothyroidism and hyperthyroidism, respectively (3–6). The mechanism is thought to be linked to molecular mimicry between *H. pylori* antigens and thyroid constituents (4, 6). Furthermore, the association is stronger in patients infected with CagA-positive strains that may be linked to cross-reactivity between antibodies against *H. pylori* CagA protein and follicular cells of the thyroid gland (6). The fact that *H. pylori* eradication leads to decreasing levels of thyroid autoantibodies reinforces the putative role of the infection in ATD (7). However, only one study evaluated *H. pylori* status in children with ATD (8). In addition, we are not aware of studies on *H. pylori* infection in children with other thyroid diseases.

Because the high daily requirement of levothyroxine for the treatment of hypothyroidism has been demonstrated in *H. pylori*-positive adults (9, 10) it could be expected in childhood. Furthermore, in as much as bacterium eradication leads to a reduction of levothyroxine requirement in adults (9, 10), it would also benefit children.

Therefore, our aim was to investigate the association of thyroid dysfunction with *H. pylori* infection in childhood.

## Materials and Methods

Looking for poor outcomes that could be associated with *H. pylori* infection in childhood, a prospective cross-sectional study was conducted in a cohort of 142 children and adolescents who were randomly assessed. Among them, 106 had congenital hypothyroidism (CH) diagnosed in the public neonatal screening program of the state of Minas Gerais, and 36 children were followed up for thyroid diseases in a Pediatric Endocrinology service of the Hospital das Clínicas - Universidade Federal de Minas Gerais (UFMG). The patients were of similar low socioeconomic level and those with hypothyroidism were under strict control on levothyroxine replacement, according to their bone surface area. They also were asymptomatic in respect to gastric complaints.

Patients with either chronic disease, such as those linked to gastrointestinal absorption alteration, or other autoimmune diseases were not included. The exclusion criteria also included the use of antimicrobial and anti-inflammatory drugs, proton pump inhibitors, and H2 receptor antagonists up to 30 days before the assessment.

The study was approved by the Ethics Committee of the Institution (CAAE-06276712.4.0000.5149) and children, whenever possible, and their legal guardians signed the informed consent.

*H. pylori* status was evaluated by the ^13^C-Urea Breath Test (IRIS®, Wagner, Analysen Technik Bremen, Germany). Serum thyroid-stimulating hormone (TSH), free tetraiodothyronine **(**T4), and free triiodothyronine **(**T3) were assessed using the ICMA, UniCel® DxI - Beckman Coulter, being the reference value of.34 to 5.6 μUI/ml, 0.54 to 1.24 ng/dl, and 2.5 to 3.9 pg/ ml, respectively. The presence of antithyroid antibodies by antithyroperoxidase (ATPO), antithyroglobulin (antiTg), and anti-TSH receptor (TRAb) was evaluated either by ICMA or ECLIA, Modular E® - Roche, being the reference values of < 9 UI/mL, < 4,11 UI/mL, and < 1,75 UI/mL, respectively.

Data were analyzed by the SPSS statistical software package version 20.0 (Chicago, IL, USA). In the logistic models, all the variables with a *p*-value of 0.25 or less in the univariate analyses were included in the full model of logistic regression. Odds ratio (OR) was used as an estimate of the risk. In rare instances when Cornfield estimates of 95% CI were inaccurate, exact limits were calculated instead. The Hosmer-Lemeshow goodness-of-fit test was used to evaluate the fit of the models.

For other comparisons, either the Kolmogorov-Smirnov test or Shapiro-Wilk test based on the number of each sub-group was used to assess the normality of the data. Two-tailed student's t-test or Mann-Whitney U test as well as χ^2^ with Yates' correction or Fisher's exact test were employed as indicated. A *p*-value ≤ 0.05 was considered statistically significant.

## Results

A hundred and six children had CH (55.7% female, aged 8.79 ± 5.17 years) and 36 had ATD (61.1% female, 13.72 ± 3.72 years old), among them, 27 with HT and 9 with GD.

The prevalence of *H. pylori* infection was of 19.44% in the ATD children. Neither the gender (*p* = 0.53, OR = 0.74, 95%CI = 0.11–5.01), nor the mean ± SD serum levels of TSH (5.52 ± 7.66 vs. 6.61 ± 5.03, *p* = 0.41), T3 (4.27 ± 0.68 vs. 4.27 ± 0.26, *p* = 0.40), T4 (1.20 ± 0.33 vs. 1.29 ± 0.51, *p* = 0.66), ATPO (76.69 ± 97.48 vs. 78.72 ± 101.73, *p* = 0.89), anti-Tg (10.35 ± 12.14 vs. 6.5 ± 9.18, *p* = 0.54), and TRAb (4.40 ± 7.82 vs. 2.43 ± 4.57, *p* = 0.41) was associated with the infection (*H. pylori*-negative vs. *H. pylori*-positive status, respectively). The mean age ± SD was higher (*p* = 0.05) in the *H. pylori*-positive (16.00 ± 3.26;) than in the *H. pylori*-negative (13,15 ± 3.66) children.

Thirty-seven (34.90%) children with CH were infected with *H. pylori* (10.74 mean age ± 4.33 SD) and 69 (65.10%) were *H. pylori*-negative (7.71 mean age ± 5.26 SD). The mean T3 serum level (3.59 ± 0.84) was significantly lower (*p* = 0.001) in the infected children than in those free from the infection (3.95 ± 0.89), association that remained in the multivariate analysis of the logistic model, independently of the other variables (Table 1). The mean ± SD levels of ATPO (1.49 ± 0.27), the antiTg (2.61 ± 0.32) and TRAb (0.43 ± 0.28) were in the reference values.

**Table 1:** *H. pylori*-positive compared with *H. pylori*-negative status in children with CH.

**Covariate**	**Univariate analysis**	**Multivariate analysis**		
	**P**	**OR**	**95% CI**	**P**
Age	0.002	1.10	0.99–1.20	0.09
Gender	0.46	–	–	–
FT3 pg/mL	<0.001	0.26	0.30–0.98	0.03
FT4 ng/dL	0.30	–	–	–
TSH μUI/mL	0.03	1.05	0.97–1.14	0.25

*The Hosmer-Lemeshow test showed a good fit of the model (10 steps; 8 degrees of freedom; p >0.25). CH, Congenital Hypothyroidism; ATD, Autoimmune Thyroid Disease; FT3, free triiodothyronine; FT4, free tetraiodothyronine; TSH, thyroid-stimulating hormone*.

The children did not require an increased daily dose of thyroxine, independent of being *H. pylori*-positive or negative.

When the diseases were compared, the *H. pylori* status, T3 levels, and age were selected in the univariate analysis. In the multivariate analysis, the T3 level (3.82 mean ± 0.88 SD vs. 4.26 mean ± 1.76 SD, respectively) and the age (8.79 mean ± 3.72 SD vs. 13.72 mean ± 5.14 SD, respectively) remained negatively associated with CH and *H. pylori*-positive status positively associated with CH (Table 2). The mean levels of ATPO, antiTg, and TRAb were significantly lower in the CH than in the children with ATD (*p* < 0.001 for all).

**Table 2:** Host variables associated with CH in comparison with ATD children.

**Covariate**	**Univariate analysis**	**Multivariate analysis**		
	**P**	**OR**	**95% CI**	**P**
Age	0.002	0.68	0.58–0.79	<0.001
Gender	0.78	–	–	–
HP+	0.11	3.20	1.10–9.60	0.03
FT3 pg/mL	0.13	0.36	0.19–0.67	0.001
FT4 ng/dL	0.48	–	–	–
TSH μUI/mL	0.39	–	–	–

*The Hosmer-Lemeshow test showed a good fit (10 steps; 8 degrees of freedom; p >0.25). +, positive; CH, Congenital Hypothyroidism; ATD, Autoimmune Thyroid Disease; FT3, free triiodothyronine; FT4, free tetraiodothyronine; TSH, thyroid-stimulating hormone*.

## Discussion

In this cross-sectional study, we evaluated the effects of *H. pylori* infection in children with thyroid disease with an emphasis on CH disease which affects 1:2,000–2,500 newborns (11). There is a consensus that the prevalence of thyroid dysfunction varies according to age, sex, geographical factors, and iodine intake.

Although *H. pylori* infection, especially with CagA-positive strains, has been demonstrated to be associated with ATD in adults (3–6), there is only one study evaluating thyroid disease in children (8). The authors observed an association between the ATD disease and *H. pylori*-positive status, but only concomitantly with the presence of the HLA-DRB1^*^0301 allele, a genetic marker of autoimmunity. No association was seen in the presence of *H. pylori* positivity/HLA-DRB1^*^0301 allele negativity as well as *H. pylori* negativity/HLA-DRB1^*^0301 allele positivity. Therefore, this significant interaction between HLA-DRB1^*^0301 genotype and *H. pylori* infection supports the role of simultaneous genetic and environmental factors in sustaining the thyroid disease in children. Similarly, we did not observe an association between ATD and *H. pylori* infection in children, considering that the frequency of *H. pylori*-positive status was low, but as expected, it increased with age. Therefore, we may speculate that the role of *H. pylori* in ATD differs between children and adults. In fact, the disease in childhood is at an early stage, and either a longer time of infection or more than one risk factor may be necessary to develop the disease, such as the presence of the HLA-DRB1^*^0301 allele observed in the study of Larizza and colleagues. The interaction of a genetic and an environmental factor seems to trigger to the development of ATD in childhood (12).

Reinforcing our results, although the hypothesis that the link between *H. pylori* infection and thyroid autoimmunity has been attributed to cross-reactivity between antibodies against *H. pylori* and thyroid constituents, we did not observe differences in the ATPO, anti-Tg, and TRAb levels between the *H. pylori*-positive and -negative children. In a study of the Czech general population (13), the authors observed that persons older than 18 years showed a higher occurrence of antibodies against *H. pylori* and of ATPO, which was not confirmed in the age group younger than 18 years. The lack of association of antibodies with anti-thyroid levels and *H. pylori* infection in children may be due to the fact that it may require a longer time of infection to significantly increase the levels of the autoantibodies.

Because we are unaware of studies evaluating the association between *H. pylori* infection and thyroid dysfunction of other etiology in children, we also evaluated children with CH. To the best of our knowledge, this is the first study demonstrating a high prevalence of *H. pylori* infection in children with CH (34.90%). Although the prevalence of HP infection varies among countries and regions of a country, notably, in the same Brazilian city, Belo Horizonte, a similar *H. pylori* prevalence (37.59%) was demonstrated in a cohort of children submitted to gastroduodenal endoscopy due to gastrointestinal symptoms (14, 15). In another study from northeast Brazil, the *H. pylori* prevalence was reported to be 21.5% in asymptomatic younger children six to 30 months of age (16). Taking together all the data, the differences between children with CH and those with ATD in the present cohort are remarkable. When the diseases were compared, we observed a significantly higher prevalence of *H. pylori* infection among younger children with CH than in those with ATD (19.44 %). It was unexpected because the infection increases with increasing age as we observed in the children with ATD, who were older than those with CH. In addition, it may not be attributed to differences in socioeconomic level.

The significant association between *H. pylori* and CH suggests that the disease could be a predisposing factor to *H. pylori* infection allowing the gastric colonization of the microorganism.

Congenital hypothyroidism is a functional glandular disturbance, due to congenital thyroid dysgenesis or dishormonogenesis (11). Impairment in the development of the immune system has been demonstrated in mice carrying a mutation (hyt) that results in the phenotypic expression of congenital hypothyroidism (17). Whether some degree of immune response impairment is associated with the human congenital glandular disturbance, it would predispose an increased risk of *H. pylori* infection in childhood.

Furthermore, surprisingly, we observed that serum T3 was lower in *H. pylori-*positive than in *H. pylori-*negative children without differences in the levels of TSH and T4, indicating that the infection leads to an impairment in the thyroid hormonal balance, but not in the hypothalamic-pituitary-thyroid axis function.

Euthyroid Sick Syndrome (ESS) is the term used to identify abnormal thyroid hormone levels in the absence of pituitary or thyroidal dysfunction. According to Chopra et al. (18), the patterns of abnormalities of thyroid hormone levels in ESS are classified into four major types. The most common type, namely low T3 syndrome, was defined as a condition in which T3 is decreased, but T4 and TSH levels remain unaltered as we observed in the group of *H. pylori*-positive children. Variable patterns in serum thyroid hormone concentrations have currently been described as non-thyroidal illness syndrome (NTIS) and may probably occur in any severe illness (19).

Early studies pointed to impaired conversion of the inactive pro-hormone T4 to the biologically active metabolite T3 by the 5-monodeiodinase in the peripheral tissues. The exact cause of changes in thyroid hormone levels observed in NTIS remains controversial and undermined, but would be linked to inflammatory pathways, including cytokine inhibition of the 5-monodeiodinase enzyme. Tumor necrosis factor-alpha (TNF-α) exerts various effects on many cell types. Administration of TNFα to rats decreases hepatic 5'-deiodinase activity (20) and TNF-α has been implicated in the pathogenesis of the low triiodothyronine syndrome in non-thyroidal illness and humans (21, 22). *H. pylori* infection in children is associated with increased inflammatory cytokines release, especially those linked to the innate immune response, among them TNF-α which is increased in children more than in adults with the infection (23).

Of note, in contrast to that observed in adults, the *H. pylori*-positive children with hypothyroidism did not require an increased daily dose of thyroxine. In fact, in adults, impairment of absorption has been associated with an altered acid environment of the stomach due to the infection as well as long-term treatment with proton-pump inhibitors, which are uncommon conditions in children.

Although our study does have some limitations such as the ATD sample size, which could have made the results biased, as well as the incomplete characterization of the bacterium CagA status, the rigorous experimental protocol, and the data analysis strengthen our results. It has to be mentioned that the children were prospectively evaluated and the *H. pylori* status was investigated by a direct test that detects only ongoing infection in contrast to the *H. pylori* infection detection by the presence of antibodies against the bacterium. Of note, serological detection of *H. pylori* antibodies is not useful to discriminate between past and ongoing infections. ^13^C-UBT, in addition to the stool antigen test, is currently considered the preferred non-invasive method to detect *H. pylori* infection (16, 24). ^13^C-UBT, besides being validated for Brazilian children, is highly sensitive and specific for the detection of *H. pylori* in children of all ages (16, 24). It is well recognized that children may gain and lose *H. pylori* infection and the serum levels of the antibodies against the bacterium may persist after spontaneous loss.

Taken together, our findings indicate that *H. pylori* infection is not associated with ATD in children, but its prevalence is high in young patients with CH and leads to impairment in the T3 metabolism. In addition, children with hypothyroidism infected by *H. pylori* did not require an increase in the daily dose of thyroxine.

To conclude, we would like to point out that as *H. pylori* infection is highly widespread and the infection is mainly acquired in childhood, in addition to the fact that the prevalence of CH is also not negligible, additional studies are required to confirm our results and to identify the involved mechanisms.

## Data Availability Statement

The original contributions presented in the study are included in the article/supplementary material, further inquiries can be directed to the corresponding author/s.

## Ethics Statement

The studies involving human participants were reviewed and approved by Ethics Committee of the Universidade Federal de Minas Gerais (UFMG) – CAAE-06276712.4.0000.5149. Written informed consent to participate in this study was provided by the participants' legal guardian/next of kin.

## Author Contributions

Conceptualization and writing—original draft preparation: IS, LM, and DQ. Methodology: LM and DQ. Formal analysis: IS and DQ. Writing—review: DQ. All authors contributed to the article and approved the submitted version.

## Funding

This study was partially supported by the Laboratory of Research in Bacteriology, Faculdade de Medicina, Universidade Federal de Minas Gerais.

## Conflict of Interest

The authors declare that the research was conducted in the absence of any commercial or financial relationships that could be construed as a potential conflict of interest.

## Publisher's Note

All claims expressed in this article are solely those of the authors and do not necessarily represent those of their affiliated organizations, or those of the publisher, the editors and the reviewers. Any product that may be evaluated in this article, or claim that may be made by its manufacturer, is not guaranteed or endorsed by the publisher.
